# Normal Tissue Dosimetry of Intraperitoneal ^224^Ra Microparticle Therapy: Data from First-in-Human Studies in Patients with Peritoneal Metastases

**DOI:** 10.2967/jnumed.125.271925

**Published:** 2026-10

**Authors:** Simen Rykkje Grønningsæter, Johan Blakkisrud, Monika Kvassheim, Sara Westrøm, Anne-Kirsti Aksnes, Ayca Løndalen, Mona-Elisabeth Revheim, Øyvind Sverre Bruland, Lars Tore Gyland Mikalsen, Yun Wang, Stein Gunnar Larsen, Caroline Stokke

**Affiliations:** 1 Department of Physics and Computational Radiology, Division of Radiology and Nuclear Medicine, Oslo University Hospital, Oslo, Norway;; 2 Department of Physics, University of Oslo, Oslo, Norway;; 3 Faculty of Medicine, University of Oslo, Oslo, Norway;; 4 Oncoinvent Solutions AS, Oslo, Norway;; 5 Department of Diagnostic Imaging, Innlandet Hospital Trust, Elverum-Hamar, Norway;; 6 Intervention Centre, Division of Technology and Innovation, Oslo University Hospital, Oslo, Norway;; 7 Department of Oncology, Norwegian Radium Hospital, Oslo University Hospital, Oslo, Norway;; 8 Institute of Clinical Medicine, University of Oslo, Oslo, Norway; and; 9 Department of Surgical Oncology, Oslo University Hospital, Oslo, Norway

**Keywords:** normal tissue dosimetry, ^224^Ra, α-emitter therapy, peritoneal metastasis, biokinetic modeling

## Abstract

Two early-phase clinical studies evaluated α-emitting ^224^Ra adsorbed to calcium carbonate microparticles ([^224^Ra]CaCO_3_-MP). Patients with peritoneal metastases from ovarian or colorectal cancer underwent complete cytoreductive surgery 2 days before intraperitoneal injection of [^224^Ra]CaCO_3_-MP. The aim of this work was to assess biodistribution and estimate absorbed doses to normal organs and tissues in a dosimetry cohort. **Methods:** Three patients with ovarian cancer and 6 patients with colorectal cancer were administered 7 MBq of [^224^Ra]CaCO_3_-MP intraperitoneally. Blood was sampled at 5 time points. ^224^Ra and ^212^Pb in blood were estimated by γ-counter measurements of photons originating from ^212^Pb and its daughters. Biexponential curves fit to ^224^Ra and ^212^Pb blood activities were combined with biokinetic models from International Commission of Radiation Protection (ICRP) publications 137 and 151 to estimate absorbed doses. γ-camera images were acquired to evaluate biodistribution. Additionally, a sensitivity analysis was performed to investigate the effect of deviation in ICRP transfer coefficients. **Results:** The highest absorbed dose was to the endosteal bone surface, receiving up to 0.85 Gy/7 MBq, followed by kidneys, liver, and red bone marrow receiving up to 0.35, 0.28 and 0.14 Gy/7 MBq, respectively. In the sensitivity analysis, the range was 0.18–2.0, 0.090–0.96, 0.083–0.62, and 0.042–0.31 Gy/7 MBq for the 4 organs, respectively. Besides radioactivity in the content of the large intestine at later time points, no visibly distinguishable uptake was assessed outside the peritoneal cavity on γ-camera images, supporting the modeling results. **Conclusion:** Based on biokinetic modeling combined with measured activity in patient blood samples, no patient received absorbed doses exceeding 0.85 Gy/7 MBq for any organ. Combined with clinical reports, and taking the potential range in absorbed doses given by the sensitivity analysis into account, the absorbed doses to the investigated tissue are likely below the relevant range of normal tissue complication probabilities for this treatment.

## Visual Abstract

**Figure:**
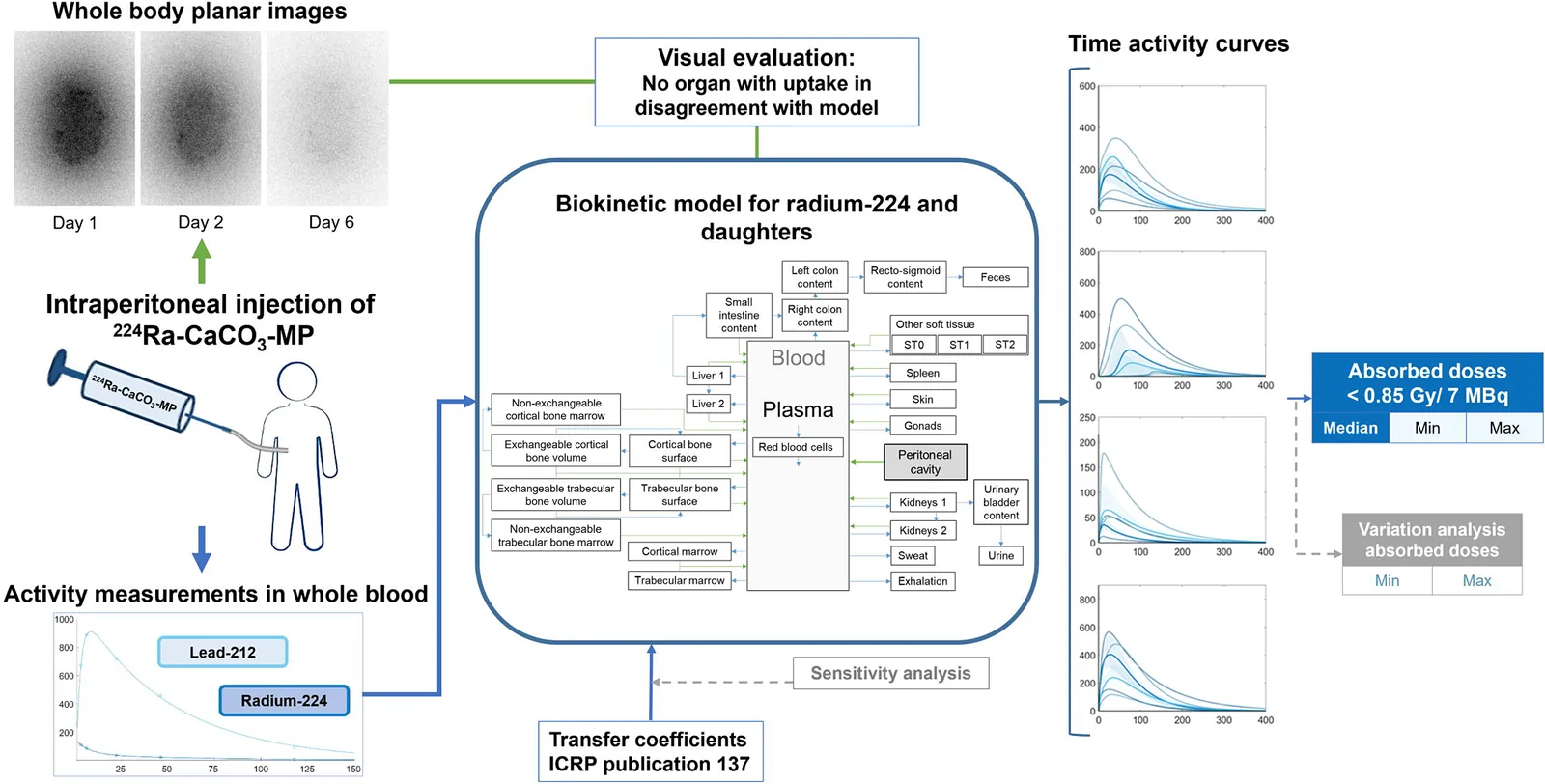

Peritoneal metastases arise from several malignancies, most commonly in ovarian and colorectal cancers. Despite comprehensive treatment strategies, including cytoreductive surgery combined with systemic or hyperthermic intraperitoneal chemotherapy, patients remain at high risk of disease recurrence (1,2), largely attributable to residual microscopic tumor deposits (3). Peritoneal recurrence is associated with a markedly worse prognosis compared with other patterns of disease relapse (2). ^224^Ra adsorbed to calcium carbonate microparticles ([^224^Ra]CaCO_3_-MP, or Radspherin; Oncoinvent) is being developed as an adjuvant treatment after complete cytoreductive surgery with the rationale to irradiate remaining micrometastases and free-floating cancer cells after the surgical procedure (4). Two studies of [^224^Ra]CaCO_3_-MP injected intraperitoneally have been performed, one phase 1 study including patients with platinum-sensitive recurrent ovarian cancer and one phase 1/2a study including patients with colorectal cancer (5,6).

The mechanism of action for [^224^Ra]CaCO_3_-MP is to deliver short-range α-radiation to the volume and linings of the abdominal cavity. ^224^Ra has 4 α- and 2 β-decays in its decay chain (Fig. 1), depositing a total mean energy of 26.5 MeV from α-particles. In vitro studies have shown that both ^224^Ra and ^212^Pb primarily stay adsorbed to the calcium carbonate microparticles (7). However, free radionuclides can potentially leave the peritoneal cavity and be transported to other parts of the body. In mice, reduced leakage of ^224^Ra and ^212^Pb from the peritoneal cavity were observed when comparing [^224^Ra]CaCO_3_-MP to ^224^RaCl_2_ (7). Biokinetic modeling can be used to calculate the spatiotemporal distribution of radionuclides and is useful to support dosimetry calculations when γ-camera imaging is challenging (8). The international committee of radiation protection (ICRP) has published biokinetic models (Fig. 2) with transfer coefficients for radium and its daughters in ICRP publications 137 and 151 (9,10), which have previously been applied in modeling studies of both ^224^RaCl_2_ (11) and ^223^RaCl_2_ (12).

**FIGURE 1.**
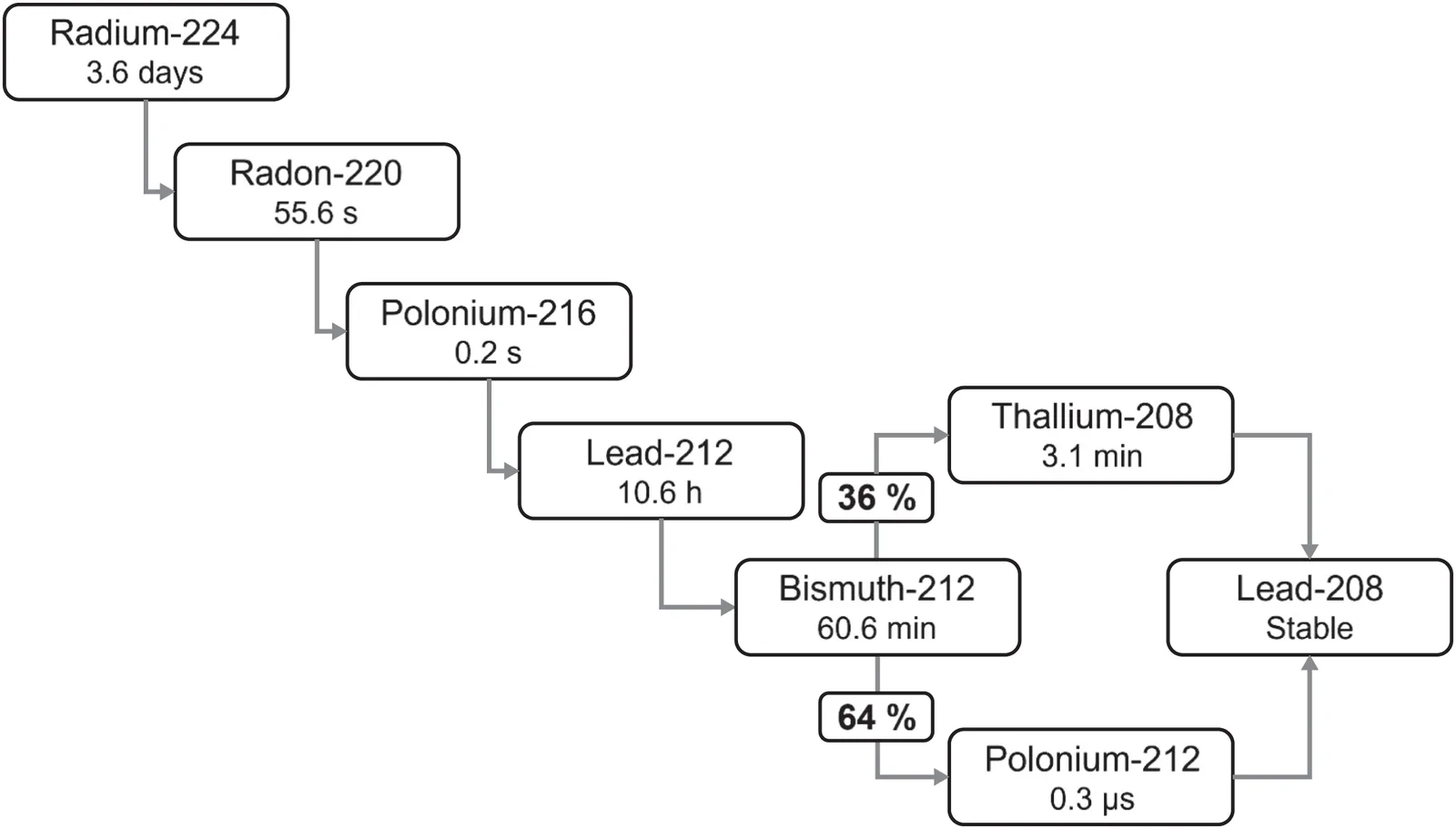
Decay chain of ^224^Ra with daughters, indicating half-lives and type of decay (15). Radiations and energies are shown in Supplemental Files 1.

**FIGURE 2.**
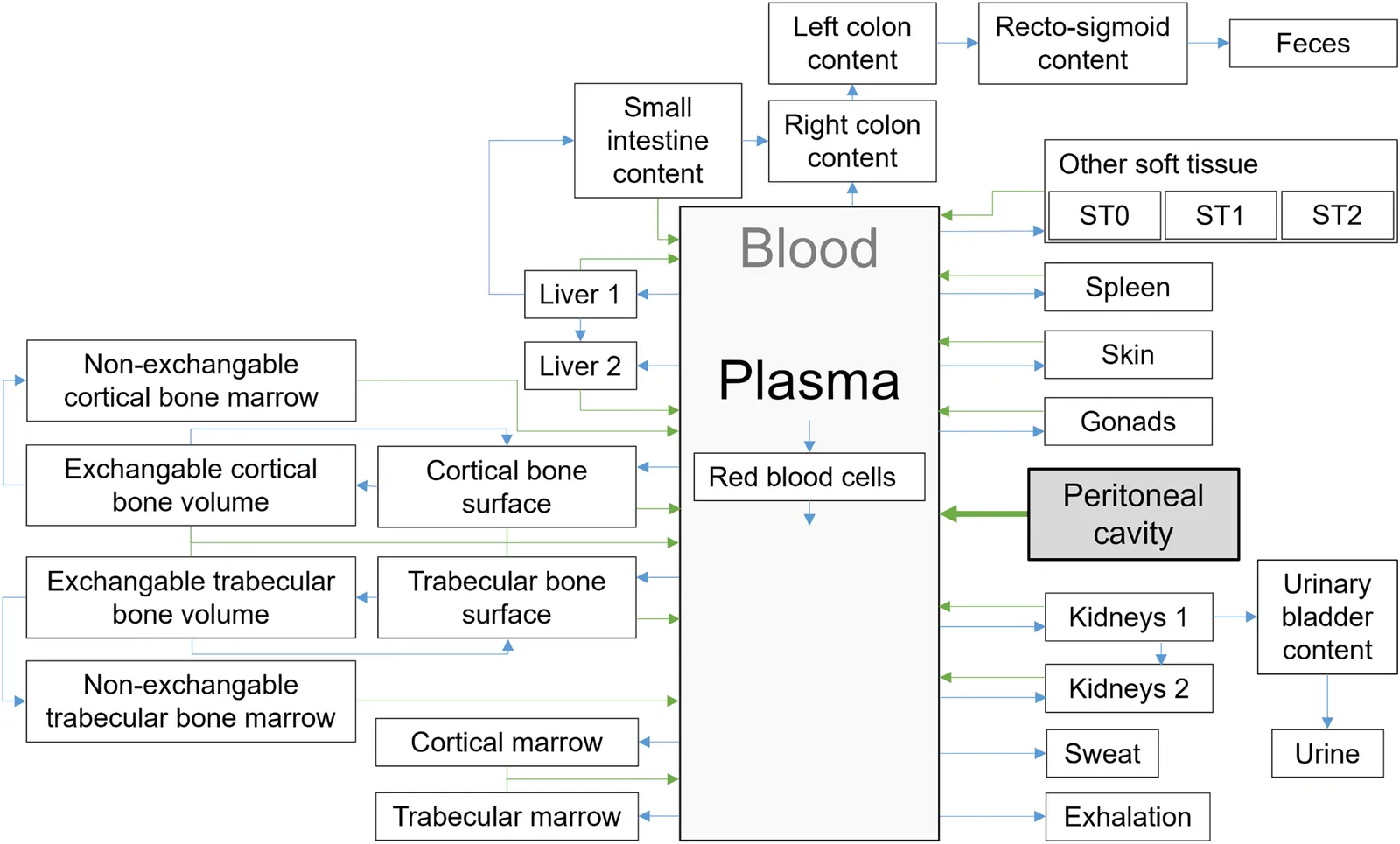
Radionuclide distribution pathways illustrated with arrows indicating radionuclide-specific transfer coefficients. Blue arrows denote transport from plasma, whereas green arrows indicate transport back to plasma. ST = soft tissue.

The primary aim of this work was to estimate the biodistribution and absorbed doses to the normal tissues defined as target organs in ICRP publication 133 (13) after intraperitoneal administrations of [^224^Ra]CaCO_3_-MP. Organ-level dosimetry was based on ICRP biokinetic modeling combined with measured activity in patient blood samples, assumed to reflect free ^224^Ra and ^212^Pb having entered systemic circulation from the peritoneal cavity. As the ICRP transfer coefficients are population-based (9,14), a secondary aim was to perform a sensitivity analysis to explore the potential range of absorbed doses.

## MATERIALS AND METHODS

### Patient Cohorts and Biodistribution Measurement Protocol

Cohorts from 2 clinical studies of [^224^Ra]CaCO_3_-MP were included: 3 patients with peritoneal metastasis from recurrent ovarian cancer (NCT03732768) and 6 with peritoneal metastasis from colorectal cancer (NCT03732781). The studies were approved by the regional ethical review board. All patients gave written informed consent. All patients underwent complete cytoreductive surgery, with no visible residual disease. For patients in the colorectal cohort, cytoreductive surgery was followed by hyperthermic intraperitoneal chemotherapy.

One administration of approximately 7 MBq of [^224^Ra]CaCO_3_-MP (Table 1), in equilibrium with its daughters, containing 0.7–1 g of microparticles, was injected intraperitoneally via a peritoneal catheter 2 days postoperatively. Before injection, [^224^Ra]CaCO_3_-MP was diluted to 50 mL with Plasmalyte (Baxter). The injection was followed by bolus flushing with approximately 200 mL of Plasmalyte. Whole blood samples of at least 3 mL were collected at multiple time points postinjection for activity concentration analysis. Figure 3 summarizes the treatment, imaging, and blood sampling procedure.

**TABLE 1.** Patient Characteristics

Cohort	Sex	Injected activity of ^224^Ra (MBq)	Body weight (kg)	Height (m)	Age (y)
Ovarian origin	Female	7.10	63	1.65	55
	Female	7.00	85	1.67	56
	Female	7.01	77	1.68	79
Colorectal origin	Female	7.22	66	1.56	68
	Female	6.99	92	1.77	56
	Male	7.05	82	1.78	66
	Female	7.22	59	1.64	43
	Female	7.36	67	1.64	28
	Female	7.08	81	1.58	68

**FIGURE 3.**
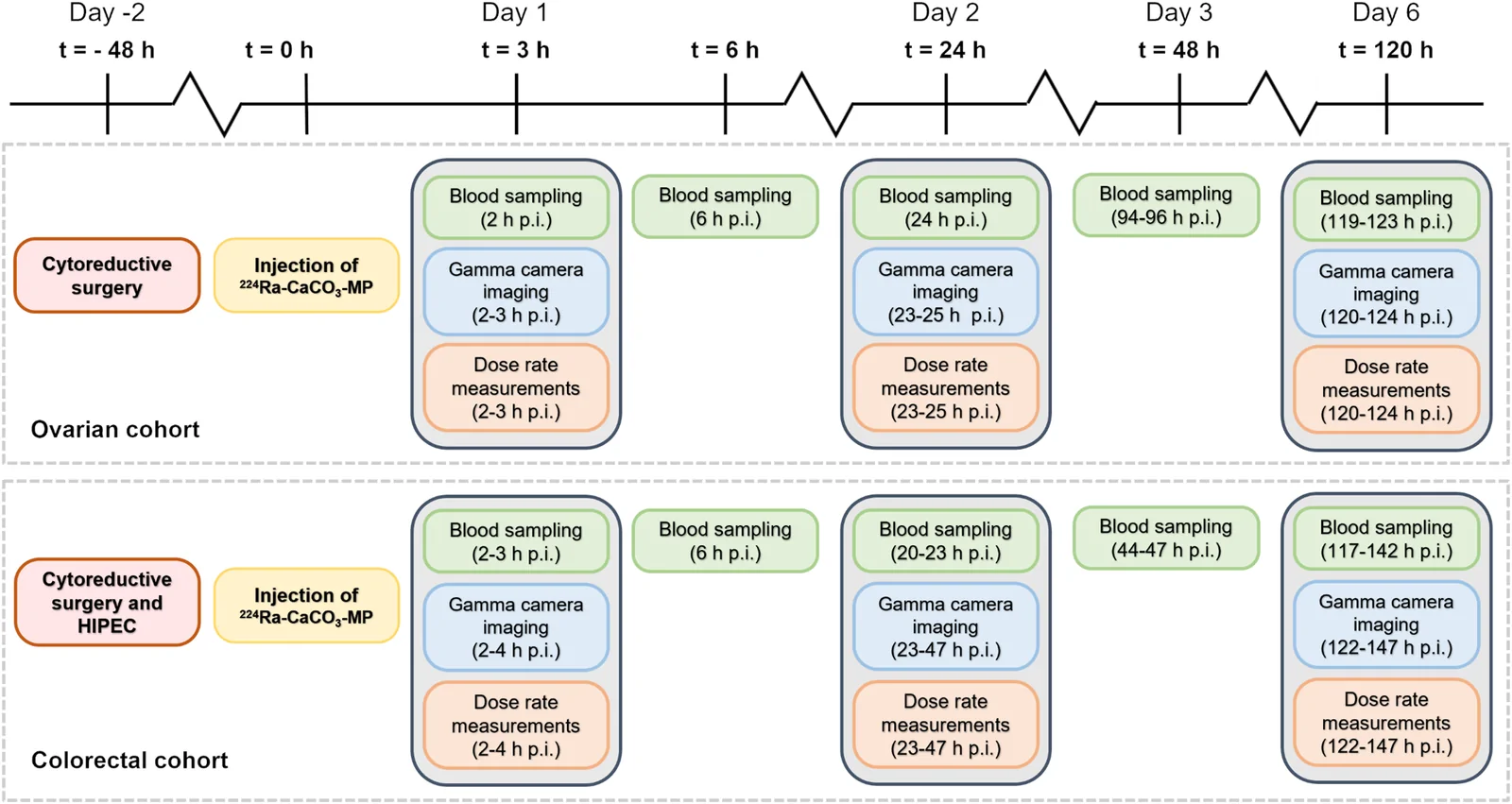
Overview of data collected for normal-tissue dosimetry study. Scheduled time points are shown in time line, whereas range of collected time points are indicated together with type of measurement. HIPEC = hyperthermic intraperitoneal chemotherapy; p.i. = postinjection.

### Activity Calculation for Blood Samples

To calculate the activity of ^224^Ra and ^212^Pb at the time of sampling, each blood sample was measured twice for 10 min with a 60–110 keV energy window on a Hidex automatic γ-counter. Details are provided in Supplemental Files 2 (supplemental materials are available at http://jnm.snmjournals.org). The first measurement was scheduled within 4 h of sampling, and the second was 24–72 h later. The signal detected at both measurement time points contains a mix of ^212^Pb present in the sample at collection or from ^212^Pb generated from decay of ^224^Ra. The Bateman equation (16) was used to calculate the activity of ^224^Ra and ^212^Pb at sampling:

$$A_{\mathrm{Ra},t_{s}}=\frac{(A_{\mathrm{Pb},t_{2}}-A_{\mathrm{Pb},t_{1}}e^{-\lambda_{\mathrm{Pb}}(t_{2}-t_{1})})e^{\lambda_{\mathrm{Ra}}(t_{1}-t_{s})}}{\frac{\lambda_{\mathrm{Pb}}}{\lambda_{\mathrm{Pb}}-\lambda_{\mathrm{Ra}}}(e^{-\lambda_{Ra}(t_{2}-t_{1})}-e^{-\lambda_{Pb}(t_{2}-t_{1})})},$$ Eq. 1

and

$$A_{\mathrm{Pb},t_{s}}=\frac{A_{\mathrm{Pb},t_{m1}}-A_{\mathrm{Ra},t_{s}}\frac{\lambda_{\mathrm{Pb}}}{\lambda_{\mathrm{Pb}}-\lambda_{\mathrm{Ra}}}(e^{-\lambda_{\mathrm{Ra}}(t_{1}-t_{s})}-e^{-\lambda_{Pb}(t_{1}-t_{s})})}{e^{-\lambda_{Pb}\left(t_{1}-t_{s}\right)}}.$$ Eq. 2

Here, *t*_1_ and *t*_2_ are time of first and second measurement, and *t*_s_ is the time of sampling. $A_{\mathrm{Ra},t_{x}}$ and $A_{\mathrm{Pb},t_{x}}$ is the activity of ^224^Ra and ^212^Pb at designated time point *t*_1_ or *t*_2_, and λ_Pb_ and λ_Ra_ are the decay constants of ^212^Pb and ^224^Ra, respectively.

### Biokinetic Modeling

A biokinetic model of ^224^Ra and its daughters radon, lead, bismuth, and thallium (Fig. 2) was created using MATLAB R2025b (MathWorks) based on transfer coefficients (Supplemental Files 3) from ICRP publications 137 and 151 (9,10). The model was solved using a time resolution of 5 s. The polonium isotopes ^216^Po (0.145 s) and ^212^Po (0.299 μs) were assumed to coincide with the mother nuclide because of their short half-lives. A gonad compartment (testes or ovaries) with separate transfer coefficients was added. Blood volume was set to 4.1 L for women and 5.6 L for men according to ICRP publication 133 (13), and a blood density of 1 g/mL was assumed. For each patient, biexponential curve fits to ^224^Ra and ^212^Pb blood measurements were used as input to the model. An initial investigation using default ICRP transfer coefficients was performed, and the whole-body activity given by the biokinetic model (excluding exhalation, excreta, urine, and feces) was compared with whole-body measurements using root mean square error. After this, explorative scaling of the set of ICRP transfer coefficients with 40%, 60%, 80%, and 120% was investigated (Supplemental Files 4). In the immediate postoperative period, peristalsis is often delayed, and models with delayed rates of intestinal function normalization for the patients were investigated (Supplemental Fig. 1). A time-dependent function of the transfer coefficient from plasma to right colon content was defined by

$${\mathrm{TC}}_{\mathrm{Pl}\to \mathrm{RCC}}(t)=\frac{1}{1+e^{b_{0}-b_{1}t}}{\mathrm{TC}}_{\mathrm{Pl}\to \mathrm{RCC}}^{\mathrm{ICRP}\;137},$$ Eq. 3

where *TC*_*Pl→RCC*_(*t*) is the time-dependent transfer coefficients describing transfer from plasma to right colon content, and ${\mathrm{TC}}_{\mathrm{Pl}\to \mathrm{RCC}}^{\mathrm{ICRP}\;137}$ is the default transfer coefficients (9), with a *b*_0_ value of 7.2 and a *b*_1_ value of 2.4. This model showed good correspondence with whole-body measurements and required only 1 ICRP transfer coefficient to be adjusted, which was in agreement with the lack of intestinal content at early time points and was therefore used for absorbed dose calculation.

### Absorbed Dose Calculation

Time–activity curves from the biokinetic model for each source organ were analytically integrated to provide time-integrated activity. Specific absorbed fraction (SAF) values from ICRP publication 133 (13) were used to calculate absorbed dose. The SAF values for α-particles, β-particles, internal conversion electrons, and photons were adapted to the energies in the decay chain of ^224^Ra by linear interpolation. An additional source organ for the peritoneal cavity was added, using the approach by Watson et al. (17) for photons containing the time-integrated activities in the peritoneal cavity compartment. These SAF values were adapted to ^224^Ra and its daughters using linear interpolation.

Except for urine, feces, excreta, and exhalation, all compartments in Figure 2 were used as source organs with the SAF values from the ICRP publication 133. The compartment “soft tissue” was assumed to correspond to muscles, and a single compartment for blood was used (including plasma and red blood cells). In total, the SAF values for the following source organs and tissues were used: blood, cortical bone surface, cortical bone volume, cortical bone marrow, gonads, kidneys, left colon content, liver, muscle, peritoneal cavity, rectosigmoid content, right colon content, skin, small intestine content, spleen, trabecular bone surface, trabecular bone volume, trabecular bone marrow, and urinary bladder content.

### γ-Camera Imaging and Probe Measurements

A planar scan of 100-cm range starting from the base of the skull was performed on a Siemens Symbia Intevo Bold, with scan speed of 5 cm/min. A 20% energy window at 240 keV was used along with medium-energy collimators. SPECT/CT settings were selected on the basis of a previous optimization project (18) using the 240-keV window with 5% adjacent scatter windows and 60 projections of 30 s length. Images were reconstructed with the vendor’s software (Flash3D) using 30 iterations and 2 subsets, with a 12-mm Gauss filter on the main energy window and a 16-mm Gauss filter on the scatter windows. Whole-body probe measurements using RadEye SX with an FHZ 514A scintillation probe (Thermo Fisher Scientific) and SmartION 2120S (Thermo Fisher Scientific) were collected before the start of each imaging procedure, as described in a previous publication (19). Monoexponential curves were fitted to the whole-body measurements of each patient and normalized to the injected ^224^Ra activity.

### Biokinetic Model Sensitivity Analysis

To analyze the impact of deviations from ICRP transfer coefficients using individual patient data, a sensitivity analysis was performed. The aim here was not to investigate the most influential parameters (20), but rather to examine the potential range of absorbed doses. Transfer coefficients were sampled from uniform distributions within 40%–120% of the ICRP values. The values of parameters *b*_0_ and *b*_1_ in Equation 3 were sampled uniformly as floating-point numbers between 1 and 10. For each patient, 100,000 different combinations of coefficients and parameters were simulated. The scenarios resulting in the highest and lowest time-integrated activities were recorded for each radionuclide and each individual organ across all patients.

### Statistics

Descriptive statistics with median and range of values were used to describe the absorbed dose parameters. Differences of parameters between cohorts were tested with the Mann–Whitney *U* test. All statistical tests were performed with MATLAB 2025b, using a significance level of 0.05.

## RESULTS

### Blood Samples

Measurements of whole blood samples are shown in Figure 4. The ^212^Pb time-integrated activity coefficients were higher for the patients in the ovarian cohort (*P* = 0.048, median values of 12.5 and 8.55 kBqh/kBq for ovarian and colorectal cohorts, respectively) and slightly lower for ^224^Ra (*P* = 0.095, median values of 0.412 and 0.552 kBqh/kBq for the same 2 cohorts).

**FIGURE 4.**
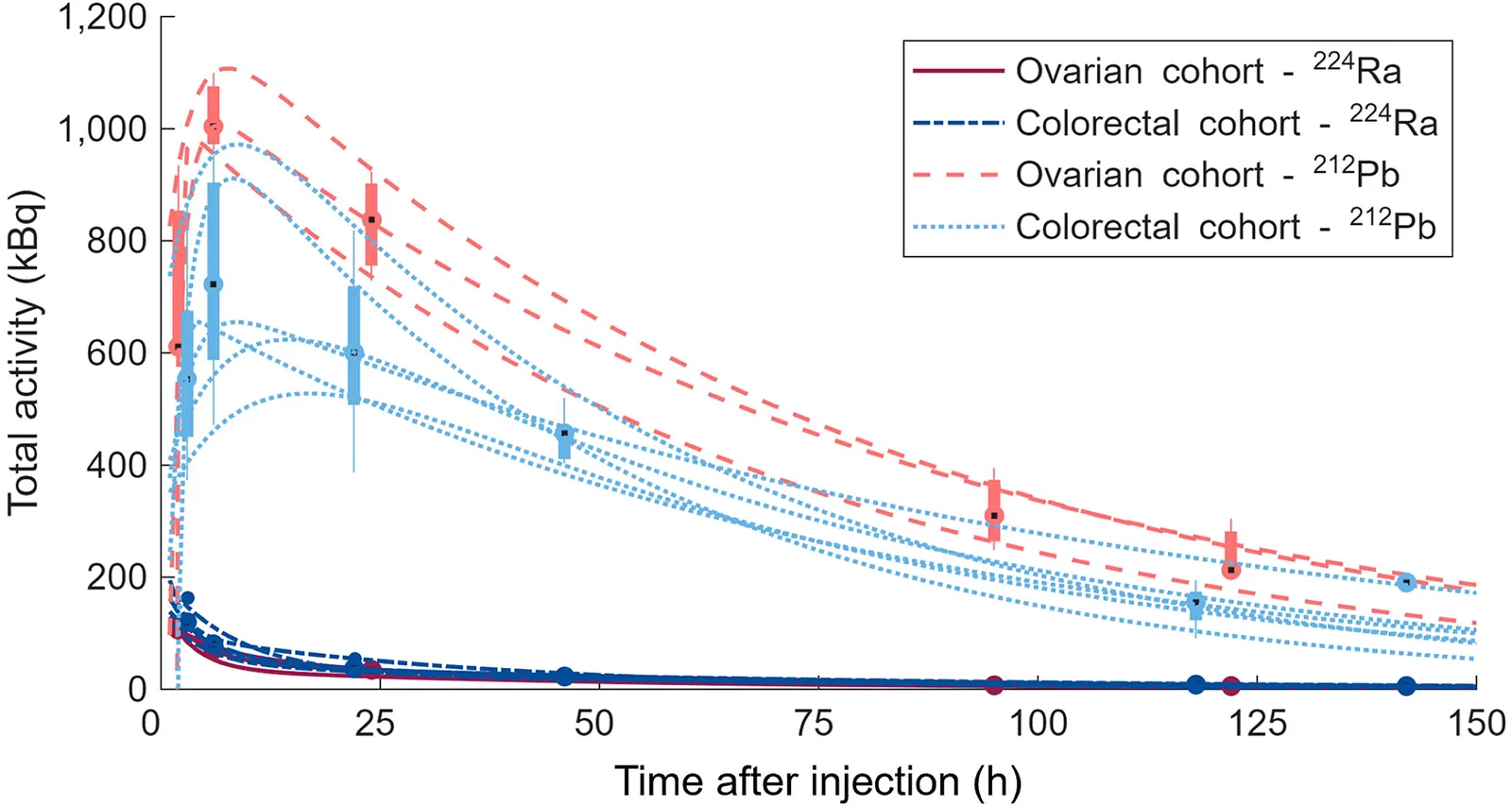
Total activity in blood (non–decay corrected). Curves represent fits to individual patient data used in biokinetic model to derive absorbed doses. Dots show mean values, thick bars show 25%–75% percentile, and thin bars show minimum–maximum.

### Modeling and Absorbed Doses

The time-dependent whole-body activities given by the biokinetic model were mainly within the minimum and maximum of monoexponential curves from probe measurements and planar γ-camera images (Supplemental Fig. 2); however, the fit improved with the delayed intestinal function model or sets of reduced transfer coefficients. Since the model with delayed intestinal function was also in accordance with low amounts of visible intestinal content for most patients on the first imaging time points, it was chosen for the absorbed dose calculation. Time–activity curves given by this biokinetic model for trabecular bone marrow and kidneys are shown in Figure 5, and a selection of additional compartments is shown in Supplemental Files 5. A summary of the absorbed doses to the included target organs are shown in Table 2. The organ receiving the highest absorbed doses was the endosteal bone surfaces, with an interpatient range of 0.52–0.85 Gy/7 MBq. This was followed by 0.22–0.35 Gy/7 MBq to kidneys, 0.21–0.28 Gy/7 MBq to the liver, and 0.10–0.14 Gy/7 MBq to red bone marrow, across all patients. Individual contributions from α, β-, and γ-emissions are presented in Supplemental Table 6. Between the patient cohorts, there were only statistically significant differences in absorbed doses to nonsource organs (*P* < 0.05) (Table 2).

**FIGURE 5.**
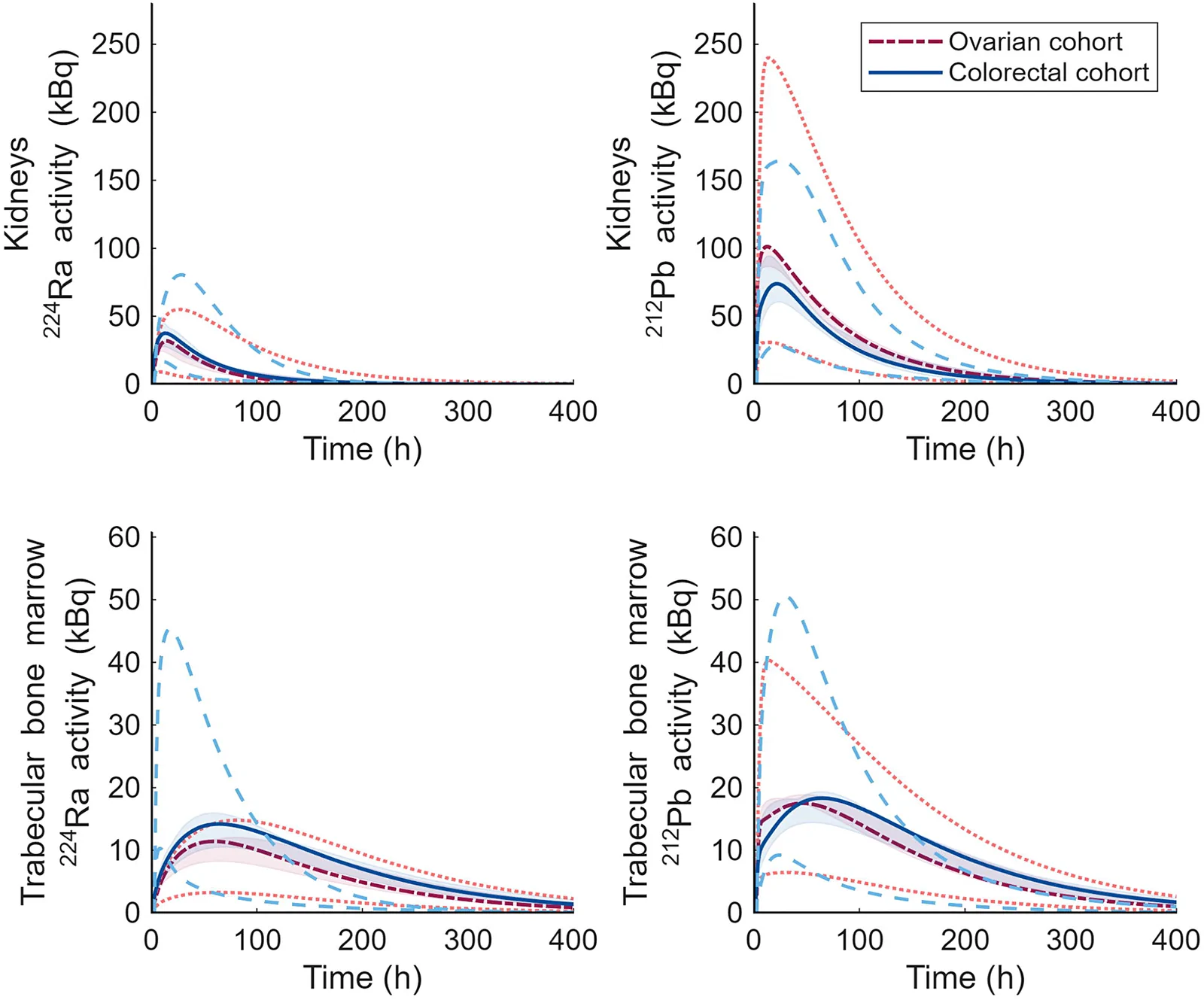
Time–activity curves of selected compartments. Thick, solid lines represent median of calculation with fixed transfer coefficients, and shaded area represents range between minimum and maximum activity values in standard scenarios. Simulated scenarios giving highest and lowest values from sensitivity analysis are shown by dot-dashed lines.

**TABLE 2.** Absorbed Doses in Gy/7 MBq for Patients Treated with [^224^Ra]CaCO_3_-MP

	Ovarian origin (*n* = 3 patients)					Colorectal cancer (*n* = 6 patients)				
	Standard coefficients			Sensitivity analysis		Standard coefficients			Sensitivity analysis	
Organ or tissue	Median	Min	Max	Lower limit	Upper limit	Median	Min	Max	Lower limit	Upper limit
Adrenals*	0.024	0.021	0.026	0.019	0.033	0.019	0.017	0.021	0.015	0.029
Alveolar interstitial*	0.066	0.052	0.067	0.045	0.079	0.045	0.039	0.055	0.033	0.068
Bronchi basal cells*	0.020	0.016	0.021	0.014	0.025	0.014	0.012	0.017	0.010	0.021
Bronchi secretory cells*	0.020	0.016	0.021	0.014	0.025	0.014	0.012	0.017	0.010	0.021
Bronchiolar secretory*	0.020	0.016	0.021	0.014	0.025	0.014	0.012	0.017	0.010	0.022
Endosteal bone surface	0.62	0.52	0.80	0.18	1.8	0.79	0.62	0.85	0.21	2.0
Esophagus*	0.023	0.018	0.023	0.016	0.029	0.017	0.014	0.019	0.011	0.024
Heart wall*	0.023	0.019	0.025	0.018	0.029	0.016	0.015	0.020	0.014	0.025
Kidneys	0.31	0.27	0.35	0.11	0.96	0.27	0.22	0.30	0.090	0.78
Left colon stem^†^	0.027	0.022	0.027	0.019	0.050	0.022	0.018	0.023	0.015	0.053
Liver	0.22	0.21	0.28	0.091	0.62	0.25	0.21	0.28	0.083	0.60
Muscle	0.021	0.020	0.027	0.011	0.062	0.025	0.017	0.027	0.0079	0.055
Ovaries^†^	0.031	0.029	0.037	0.028	0.044	0.031	0	0.034	0	0.042
Pancreas*	0.031	0.030	0.035	0.031	0.044	0.026	0.025	0.028	0.026	0.040
Rectosigmoid stem^†^	0.027	0.021	0.029	0.017	0.068	0.023	0.018	0.024	0.013	0.076
Red bone marrow	0.11	0.10	0.14	0.047	0.29	0.13	0.11	0.14	0.042	0.31
Right colon stem^†^	0.027	0.022	0.027	0.019	0.044	0.021	0.017	0.022	0.015	0.041
Skin	0.024	0.022	0.031	0.012	0.040	0.029	0.021	0.031	0.010	0.048
Small intestine stem*	0.029	0.025	0.031	0.024	0.040	0.023	0.020	0.025	0.019	0.036
Spleen^†^	0.054	0.048	0.060	0.034	0.078	0.048	0.040	0.054	0.027	0.073
Stomach stem*	0.026	0.022	0.027	0.020	0.033	0.020	0.017	0.022	0.016	0.029
Testes	0	0	0	0	0	0	0	0.02	0	0.023
Thyroid	0.015	0.012	0.015	0.010	0.019	0.011	0.009	0.013	0.0076	0.016
Uterus	0.019	0.016	0.020	0.021	0.028	0.016	0.000	0.018	0	0.028

* Significant difference between patient cohorts.

† Two patients had undergone splenectomy; all but 1 female had their gonads removed; 1 female had 1 gonad removed; 1 patient had their colon removed; and 7 patients had their rectosigmoid partially or fully removed. This was not accounted for.

Absorbed doses for organs receiving less than 0.01 Gy/7 MBq are shown in Supplemental Table 5. Standard median, minimum, and maximum represent default biokinetic model without any alterations. Here, minimum and maximum indicate individual patients. Next columns show results from sensitivity analysis, with “lower limit” and “upper limit” representing scenarios in all patients resulting in lowest and highest absorbed doses. Most patients had surgically removed either 1 or combination of organs such as ovaries, spleen, or parts of their colon (mainly the rectosigmoid colon).

### Sensitivity Analysis

Figure 5 provides the range of the sensitivity analysis, showing the curves resulting in minimum and maximum time-integrated activity for kidneys and trabecular bone marrow. Additional organs are shown in Supplemental Files 5. The variation in transfer coefficients resulted in maximum absorbed doses of 2.0, 0.96, 0.62, and 0.31 Gy/7 MBq to endosteal bone cells, kidneys, liver, and bone marrow, respectively.

### Imaging

No organs outside the peritoneal cavity had sufficient radioactivity content to be visible on neither planar images nor SPECT. The only exception was the large intestine visible at later time points for several patients (Fig. 6).

**FIGURE 6.**
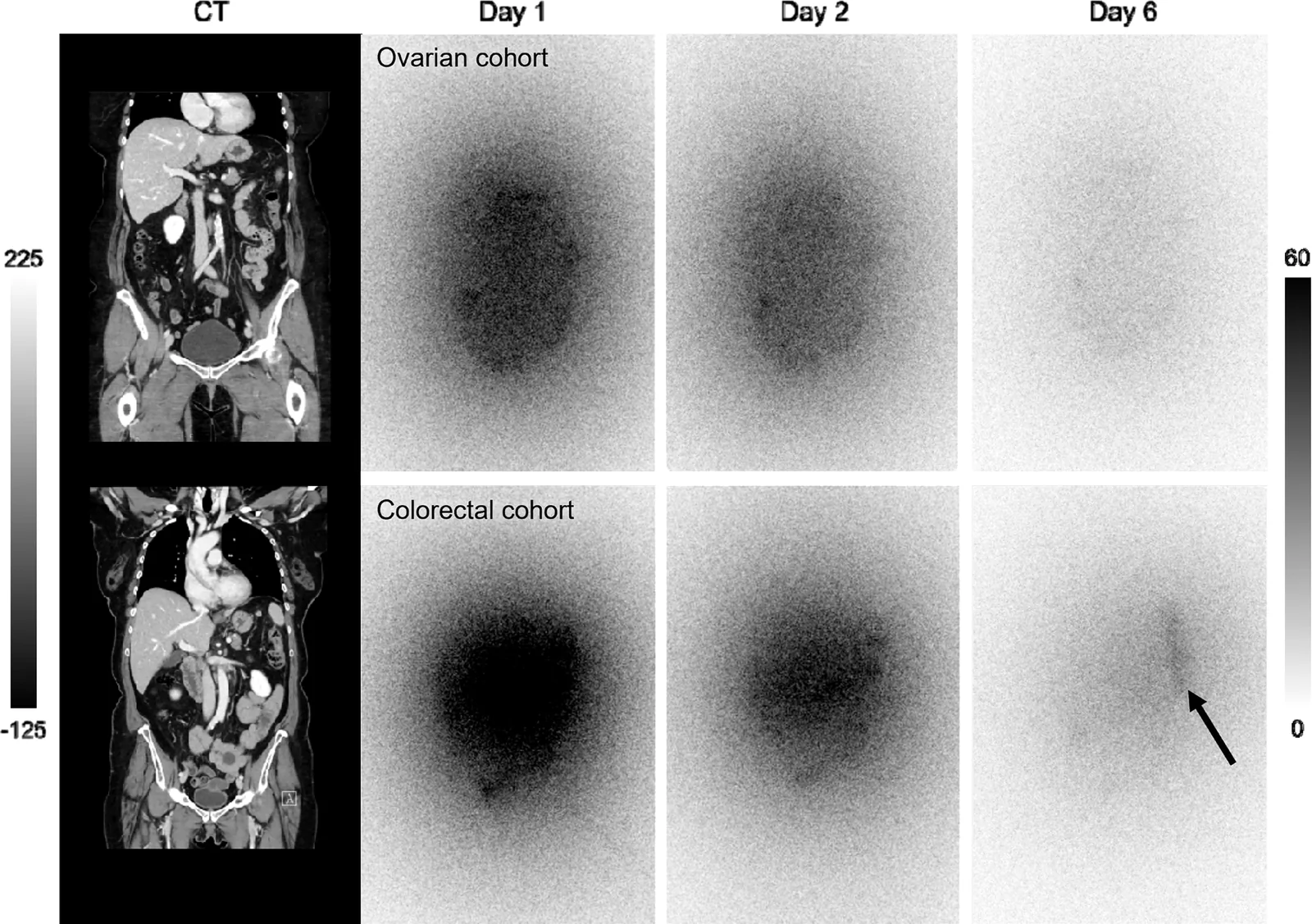
Planar anterior images of representative patient from each cohort (window, 0–60 counts used for all). For several patients, activity was assessed in contents of distal large intestine at last time point. Example can be seen in colorectal cohort patient in lower panel (arrow).

## DISCUSSION

Biokinetic models for ^224^Ra have previously been explored for treatment of ankylosing spondylitis with intravenous administration of ^224^RaCl_2_, using ICRP model 67, where the highest absorbed doses were found for bone surface, red bone marrow, and kidneys (11). Compared with our results, absorbed doses to organs such as red bone marrow, the intestinal surface, and endosteal bone surfaces were 2–5-fold lower, and liver and kidneys were a factor of 2 and 6 times higher, respectively. There are, however, key differences. Mainly, with [^224^Ra]CaCO_3_-MP, the activity was injected intraperitoneally and only a fraction of the radionuclides is assumed to leave the peritoneal cavity over time, resulting in lower normalized absorbed dose values for most tissues. For the higher absorbed doses to the liver and kidneys, this can be explained by updates from ICRP publications 67 to 137, which include refined liver and kidney compartments and revised transfer coefficients (9). Höllriegl et al. compared the distribution of ^223^Ra when using ICRP publication 67 and 137 and found that absorbed doses to kidneys were approximately 7-fold higher when using ICRP publication 137 (12). Because of the different isotopes, the results cannot be directly compared, but the trend strongly suggests that tissues with higher doses reported herein compared with Lassmann et al. (11), despite lower systemic availability of ^224^Ra, are due to the updates in ICRP model. Additionally, the ICRP publication 137, combined with ICRP publication 133, offers updates in absorbed dose estimation to the endosteum (bone surface used in ICRP publication 67), bone marrow, and the intestines (13).

Predicting effects from the estimated absorbed doses is challenging because empiric data on dose–toxicity relationships for α-emitter–based treatments are limited. α-particles are typically more damaging per unit absorbed dose than are β-particles, as intended due to the relative biological effectiveness (RBE). Biologic factors and dose heterogeneity may also differ substantially from other radionuclide treatments, hindering direct comparisons. The highest absorbed doses were found in endosteal bone surfaces, with an RBE-adjusted value of 4.2 Gy, using an RBE of 5 (21). Radiation to endosteal bone surfaces, a thin vascular membrane surrounding the bone marrow cavity, has been hypothesized to decrease bone formation and potentially also increase fracture risk (22). Such effects have been reported after 40 Gy of external beam radiation (23), and although the 2 treatment modalities cannot be directly compared, this greatly exceeds the absorbed doses found in this study. Similarly, the highest RBE-adjusted mean absorbed doses to kidneys (1.6 Gy/7 MBq) and the liver (1.3 Gy/7 MBq) were an order of magnitude below commonly used limits for radiopharmaceutical therapy (24), indicating that these organs would probably not be dose-limiting for [^224^Ra]CaCO_3_-MP. The absorbed doses to red bone marrow are potentially the most clinically relevant because of the higher radiosensitivity. Applying the RBE of 5 (21) gives an adjusted maximum dose of 0.68 Gy to red bone marrow. For comparison, the suggested toxicity limit for β-emitting targeted radiotherapy is approximately 2 Gy (25). Consistent with this result, no serious adverse events indicative of systemic toxicity, including hematologic toxicity, were reported to be related to [^224^Ra]CaCO3-MP in the dose escalation cohorts of the phase 1 studies (5,6). The organ-level dosimetric methodology only accounts for absorbed doses delivered from radionuclides in systemic circulation and photons originating from the peritoneal cavity. Hence, local electron irradiation to tissues adjacent to the peritoneal cavity is not included. Assuming homogeneous distribution within the intraperitoneal cavity, this contribution would account for less than 0.1 Gy to an intestinal membrane (defined as a section with a depth of 100–1300 µm outside a 7 MBq ^224^Ra activity layer covering 19,000 cm^2^). However, local absorbed dose variations will occur dependent on the patients’ microdistribution patterns, which are not possible to assess by γ-camera imaging. As most photons originate from ^212^Pb and its daughters, the higher blood activity concentrations in the ovarian cohort led to a significant difference in absorbed dose for several nonsource organs between the 2 patient cohorts. However, no significant differences in absorbed dose were seen for organs commonly regarded as activity-limiting.

Based on the patient γ-camera images, most activity was assessed within the peritoneal cavity. The large intestine was the only organ with distinguishable uptake, observed mainly at later time points for several patients, consistent with intestinal clearance as a known pathway of excretion for radium (12). Initially, direct comparisons between the biokinetic model and SPECT images were considered. Phantom experiments with sphere inserts were discontinued when the total activity amount corresponded to the activity amount in homogeneous water phantoms where calibration factors became unstable. At the activity concentration of 2 kBq/mL, only the 2 largest spheres were visible. This corresponds to higher amounts of ^212^Pb in organs than predicted by the biokinetic model, consistent with no additional organ uptake observed in patients. Although the imaging system has inherent performance limitations (18), this supports the model results for organs above this size. Explorative tissue quantification indicated values below the lower range of phantom activities. Adding to this, there is a risk of overestimating activities of low uptake organs (26), and as a result, absorbed dose calculations based directly on SPECT imaging were not continued.

Whole-body measurements were used to inform selection of intestinal modeling assumptions (Supplemental Fig. 2). Although uncertainties exist, especially in terms of measurement geometry, the washout appeared too fast when using default transfer coefficients (Supplemental Fig. 2E). Although the model with delayed intestinal function was chosen as the standard in this work, also to primarily use default ICRP transfer coefficients, it should be noted that the root mean square error values were fairly similar for this model and the models with downscaled sets of transfer coefficients. Since absorbed dose calculations rely on the activity in blood, absorbed dose for organs remained virtually unchanged under variations in the intestinal function model (except photon contribution). The only exception was the large intestine, where the median absorbed dose nearly doubled when the gradual increase model was omitted. However, the calculated absorbed doses to these structures remained below 0.05 Gy. Surgical removal of 1 or more organs (ovaries, spleen, or parts of the colon) was not incorporated into the biokinetic model. However, similar to the intestinal scenario, the absorbed doses to other organs would remain relatively constant, given the same observed blood activity curve, even if the transfer coefficients for a subselection of tissues were set to zero.

Biokinetic models rely on population-based values for transfer coefficients and are not intended for individual patients. Although we performed individual blood sampling, we also wanted to explore the potential range of absorbed doses resulting from variations in the transfer coefficients given by ICRP publication 137, as well as variations in Equation 3 coefficients for plasma–colon transfer by a sensitivity analysis. Variations in transfer coefficients skewed toward lower values because of the comparisons with whole-body measurements (Supplemental Fig. 2). The organs receiving the highest absorbed doses were the same using both the default transfer coefficients and the sensitivity analysis, with an average increase of 2.3 for these 4 organs. Nevertheless, the absorbed doses (2 Gy for endosteal bone cells and below 1 Gy for remaining organs) would be below values commonly associated with an increased probability of toxicity, even if adjusting for RBE.

## CONCLUSION

Estimated absorbed doses after intraperitoneal administration of 7 MBq of [^224^Ra]CaCO_3_-MP were below 0.85 Gy for all evaluated normal tissues. The aim of dosimetry investigations in early-phase studies should be to derive or confirm absorbed dose–toxicity relationships. The absorbed doses in the current study were below values commonly associated with risks for normal-tissue complications, including for organs typically regarded as activity-limiting such as kidneys and red bone marrow. Red bone marrow had the highest absorbed dose relative to assumed radiosensitivity but was still below a maximum RBE-adjusted value of 0.68 Gy (or 1.5 Gy in the sensitivity analysis exploring the range of potential absorbed doses). As no serious adverse events indicative of systemic toxicity were reported as related to [^224^Ra]CaCO3-MP in the cohorts, this supports that the absorbed doses to the investigated tissue were too low to correspond to normal-tissue complication probabilities for this treatment.

## DISCLOSURE

This study was sponsored by Oncoinvent Solutions AS. Sara Westrøm and Anne-Kirsti Aksnes are employed by and own stock/stock options in Oncoinvent. Øyvind Sverre Bruland is a cofounder, clinical consultant, shareholder, and former member of board of directors for Oncoinvent ASA. Mona-Elisabeth Revheim was a former board member of Oncoinvent AS (2023–2024). No other potential conflict of interest relevant to this article was reported.

## Notes

KEY POINTS **QUESTION:** What are the absorbed doses to normal tissues after intraperitoneal injection of [^224^Ra]CaCO_3_-MP? **PERTINENT FINDINGS:** Nine patients with peritoneal metastases from ovarian or colorectal cancer received 7 MBq of [^224^Ra]CaCO_3_-MP. Using blood sampling and biokinetic models, it was found that absorbed doses to all normal tissues studied were below 0.85 Gy. **IMPLICATIONS FOR PATIENT CARE:** The results imply that no normal tissue toxicities related to the investigated treatment are expected.
